# A new category of “Aha!” driven by touch: A grip sensation into the directional seam on a baseball

**DOI:** 10.1177/20416695231175598

**Published:** 2023-05-16

**Authors:** Yukiaki Ishida

**Affiliations:** 13143 The University of Tokyo, Japan

**Keywords:** Aha experience, tactile sense, baseball physics, point group symmetry, D_2d_ to S_4_

## Abstract

We report an “Aha!” experience which differs from conventional Aha's studied over a century in psychological science. The Aha we introduce is driven by touch instead of the visual and verbal modalities widely studied to date. It can occur when gripping a baseball, with a simple input that the red seam on the ball has a direction. Aided by a symmetry analysis and subsequent survey over literature, we show how our mental and physical representation of a baseball can change suddenly by the seam direction and unravel the factors that make the tactile sense into a joyful-and-insightful sensation. Our study sets a new category of Aha driven by touch, opens a new path to investigate the role of touch in our cognition process, reveals the seam direction as a new degree of freedom in baseball aerodynamics and pitching mechanics, and deepens the insights into throwing a baseball from our fingertips.

A baseball is not an ideal sphere but has a symbolic seam with red stitches. It is the seam of two pieces of leather cut in the shape of a peanut. The stitches make small bumps along the seam with a typical height of 0.8 mm for major league baseballs (Kensrud et al., 2015). Though tiny, the seam structure matters when gripping the ball. Finding the best way to orient the seam and place the fingers thereon continues to be the interest for pitching straight-and-fast balls or inducing effective moves such as sliders and sinkers (Nagami et al., 2015). Even a slight change in the comfort of the grip can make a salient difference in the pitch, as disputed in the 2021 season of Major League Baseball about using sticky materials when gripping (Grant, 2021; Yamaguchi et al., 2022).

The array of stitches not only makes bumps along the seam but also introduces direction in the seam (Figure 1a). Compared to the height and orientation, the direction of the seam is a structure often overlooked in baseball. Players mainly pay attention to the seam orientation but not to the seam direction when gripping a baseball (Nagami et al., 2015). In the author's survey, scientists also have left out the seam direction from consideration in the studies of aerodynamics (Alaways & Hubbard, 2001; Borg & Morrissey, 2014; Cross, 2012; Escalera Santos et al., 2019; Higuchi & Kiura, 2012; Kensrud et al., 2015; Nathan, 2008; Smith & Smith, 2021; Whiteside et al., 2016a) and pitching mechanics (Fleisig et al., 2009; Glanzer et al., 2021; Jinji et al., 2011; Kawamura et al., 2017; Kinoshita et al., 2017; Kusafuka et al., 2020; Manzi et al., 2021; Nagami et al., 2011; Nagami et al., 2015; Nasu & Kashino, 2021; Whiteside et al., 2016b), to mention a few. Even in a textbook exercise to describe the symmetry of a baseball, the seam direction is omitted (Blinder, 2004).

**Figure 1.**
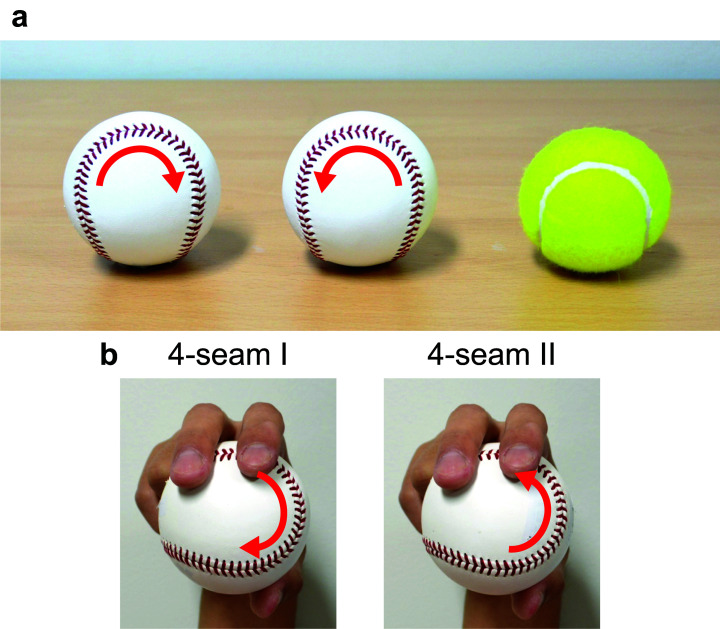
The directional seam on a baseball. (a) The two baseball seams have the same orientation but reversed direction, as indicated by the arrows. A tennis ball has no direction in the seam. (b) Subtypes in the grip to throw a 4-seam fastball. The three elite pitchers, Yoshimi, Fujikawa, and Kaneko, prefer the 4-seam I because it feels less slippery and can impart more spin (Fujikawa, 2021; Yoshimi, 2021).

A handful of elite pitchers, nevertheless, turned out to be sensible to the direction of the seam. Yoshimi (2021) and Fujikawa (2021), former professional baseball pitchers who retired in the 2020 season, recently shared their expertise through YouTube channels and disclosed their preference for one seam direction to the other when gripping to pitch a fastball. According to them, the 4-seam I grip feels less slippery than the 4-seam II and helps increase the spinning rate in the fastball pitch; see Figure 1b. Chihiro Kaneko, another elite pitcher who retired in the 2022 season, also shares the same gripping sense (Yoshimi, 2021).

We wish to elaborate on the elite sense into the seam direction from a twofold perspective. The first is from a symmetry point of view. We show that, as one becomes aware of the seam direction, the symmetry of the baseball changes from the one shared with a tennis ball to the one rarely met in our daily lives, with implications for baseball aerodynamics. Subsequently, we discuss that a unique sensation can arrive when touching the seam. It is easy to tell the seam direction through the touch owing to our fingertip sensitivity; thus, even a non-elite can acknowledge that the seam direction matters when imparting the ball from the fingertips. We elucidate that the sensational moment upon the touch meets the criteria to be identified as an “Aha!”—a phenomenal psychological experience characterized by a sudden joyful flash of insight, opening new perspectives and avenues of creativity (Topolinski & Reber, 2010). Our main argument here is that the Aha moment we report differs from the widely studied Aha's in that the driving modality is the touch. Namely, the touch sensation into the baseball seam calls for a new category in the Aha experience.

## Method: Symmetry Analysis

**Figure 2.**
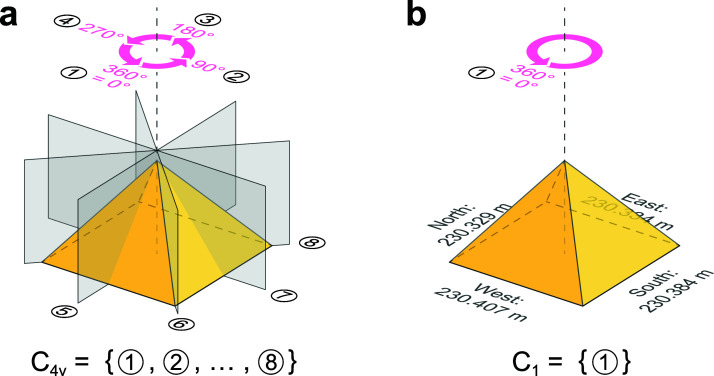
The pyramid symmetry. (a, b) Counting the symmetries of a pyramid with a square base (a) and the Great Pyramid in Egypt (b). The former has eight symmetries belonging to the point group C_4v_ while the latter has only one comprising the C_1_ group. The dimension of the base is after Dash and Paulson (2016).

The mathematical group theory for symmetries applies to the vast field of physics (Inui et al., 1990), chemistry (Cotton, 1991), and beyond (Conway et al., 2008). When applied to a three-dimensional (3D) object (Fuchigami et al., 2016), one can categorize its shape in terms of the geometric symmetries that it possesses. In theory, a geometrical operator *Ĝ* (one among the rotations, reflections, and roto-reflections) is said to be a symmetry of the 3D object if the object operated on by *Ĝ* is indistinguishable from the original. For example, the Great Pyramid in Egypt is generally known to possess eight symmetries (four-fold rotation and four mirror operations; Figure 2a) that constitute a point group called C_4v_ in the Schönflies notation (Fuchigami et al., 2016). The group, however, reduces to the simplest C_1_ if one notices the symmetry-breaking features, such as the west side of the base plane being 7 cm longer than the east side (Dash & Paulson, 2016); see Figure 2b. Here, the C_1_ point group contains only one element, the identity operator *Ê*, which leaves the object as it is.

We inspected the ball symmetries aided with round-color stickers and a mirror; subsequently, we surveyed the literature, or went through a meta-analysis, to infer the rareness of the baseball symmetry. The regulations to comply with statistical analysis standards do not apply to the inspection because symmetry analysis is free from statistical uncertainty.

## The Symmetry Analysis of a Baseball

**Figure 3.**
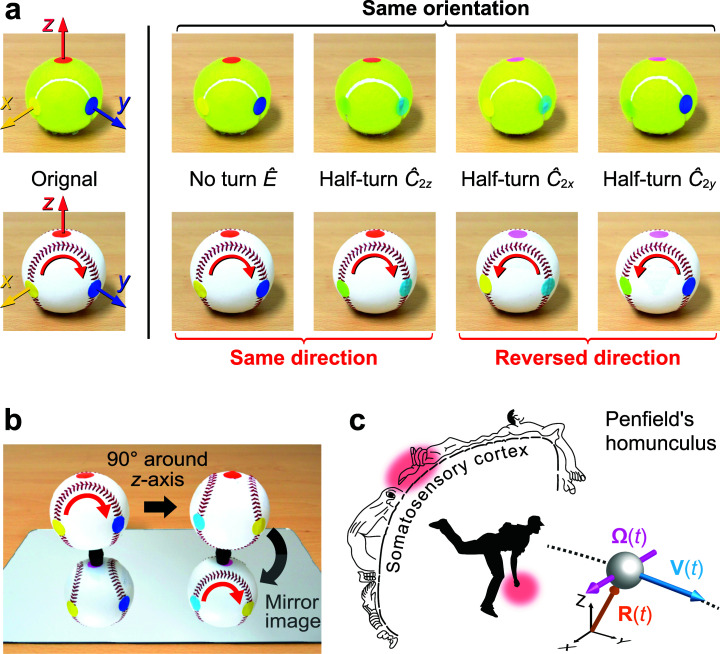
The symmetry analysis. (a) Balls before and after the half-turn rotations around the *x*-, *y*-, and *z*-axis. The round-color stickers on the ball indicate the points where the three axes pass the ball's surface. (b) The roto-reflection symmetry. The mirror image on the right is identical to the original ball on the left; thus, the *Ŝ*_4*z*_ roto-reflection is a symmetry for a baseball. (c) Sensing the seam direction with fingertips. Penfield's homunculus for the somatosensory cortex is compiled from the textbook of Anderson (2020).

Our analysis starts by searching the rotation symmetries of a baseball and a tennis ball (Figure 3a); the latter represents a baseball with no direction in the seam. Besides the identity operator *Ê*, a tennis ball has three half-turn symmetries. For a baseball, only the half-turn around the *z*-axis (*Ĉ*_2*z*_) is left as the symmetry operation; the rotations around the *x*- and *y*-axis (*Ĉ*_2*x*_ and *Ĉ*_2*y*_) are no more a symmetry because the direction of the seam reverses after the operations. Here, the *z*-axis passes through the center of the narrowest part of the peanut-shaped cover, while the *x*- and *y*-axis are perpendicular to the *z*-axis and pass through the seam on the surface; see Figure 3a.

The two half-turn rotations *Ĉ*_2*x*_ and *Ĉ*_2*y*_ are the symmetry operations for a tennis ball but not for a baseball. Therefore, when we grip a baseball and rotate it for 180° around the *x*- or *y*-axis, we end up with another grip configuration with the reversed seam (Figure 1b). There is no reason for the tribological finger-ball friction to be the same for the two grip subtypes. The way the ball spins through the air also branches into two symmetrically distinct subtypes due to the direction degrees of the seam, which applies to whatever type of pitch in principle. Ball motion studies show that the effect of the seam orientation becomes pronounced and detectable when the spinning rate is low, as in knuckleballs (Escalera Santos et al., 2019) and seam shifted wakes (Smith & Smith, 2021), and so it may be for the effect of the seam direction.

A baseball also has a roto-reflection symmetry *Ŝ*_4*z*_, a 90° rotation around the *z*-axis followed by a mirror reflection about a plane perpendicular to *z*; see Figure 3b. Operating *Ŝ*_4*z*_ twice is equivalent to *Ĉ*_2*z*_ (*Ŝ*_4*z*_^2^ = *Ĉ*_2*z*_), while *Ŝ*_4*z*_^3^ is another roto-reflection symmetry.

In total, a baseball has four geometric symmetries forming a point group called S_4_ = {*Ê*, *Ŝ*_4*z*_, *Ĉ*_2*z*_*Ŝ*_4*z*_^3^}. A tennis ball has four more symmetries, two half-turn rotations (*Ĉ*_2*x*_ and *Ĉ*_2*y*_), and two more mirror operations, which, in total, form the D_2d_ point group, as found in various exercises (Blinder, 2004; Fuchigami et al., 2016). Thus, the baseball symmetry reduces from D_2d_ shared with a tennis ball to S_4_ as one becomes aware of the seam direction (Figure 1a), similar to the pyramid symmetry reducing from C_4v_ to C_1_ due to the subtle symmetry-breaking features (Figure 2). In short, the physical representation of a baseball suddenly changes as direction sets in the seam.

It turns out that the S_4_ symmetry is rarely found in everyday objects. A piece of evidence for the rareness exists in the way the theory of symmetries is taught in the chemistry course of St. Olaf College, the United States: Students can get bonus points if they find an S_4_-symmetric object on campus, as described in the supporting information of Fuchigami et al. (2016). Here, we add a baseball as its rare realization. The fact that the baseball is rarely noticed as an S_4_-symmetric object indicates that the seam direction is indeed a structure often overlooked.

## Discussion: The Touch Sensation

Having explained that the baseball realizes the rare symmetry and directed the reader's attention to the seam direction, we wish to elaborate on the unique sensation that can arrive when gripping the ball.

As illustrated in Penfield's homunculus (Figure 3c), fingertips are a touch-sensitive part of the body (Klatzky et al., 1985; Penfield & Rasmussen, 1950). The finger touch can discern patterns as fine as 760 nm in periodicity and 13 nm in amplitude (Skedung et al., 2013). With the exquisite sensitivity of the fingertips, it is easy to tell the seam direction through the touch, even for players (such as the author) who have been unconsciously gripping it for years. It indicates that it was not the superhuman fingertip sensitivity that led the elite pitchers to discover the seam direction but their acute attentiveness during the pursuit to pitch better. Yoshimi (2021) himself says on the YouTube channel that he found it by chance. Note, a British mathematician, John H. Conway, also noticed the seam direction in the orbifold signature notation of the symmetry of things (Conway et al., 2008), presumably due to his attentiveness to keeping the mathematical rigor.

Surprisingly, it is also easy to follow the elite sense into the grip sub-types (Figure 1b). In the author's case, the 4-seam I grip indeed felt less slippery than 4-seam II, and it was immediately acknowledged that the former grip should be more effective in imparting a higher spin rate in the fastball pitch. After more than 30 years of unconsciousness, the seam direction suddenly became a significant and unforgettable structure. Further insights naturally follow: Always throwing from the 4-seam I grip will result in a consistently better pitch; some may wish to quantify the spinning rate and speed of the fastballs imparted from the two grip types with modern baseball-tracking devices.

In psychological science, an “Aha!” is described as a moment when insight comes with a sudden surprise and ease, followed by a grateful feeling and confidence in truth (Topolinski & Reber, 2010). The tactile cognition into the seam direction fully meets the four characteristics of “Aha!”: (1) It arrives immediately, owing to our fingertip sensitivity. (2) It easily changes our mental representation of the seam. (3) It is gratifying, as the sense opens a pathway to pitch better. (4) It is true, as seen in our symmetry analysis. We thus allocate the first-time tactile cognition into the seam direction as an “Aha!” moment.

The prerequisite for the touch cognition into the seam direction to become an Aha is that pitchers throw the ball from their fingertips, which is an organ endowed with expert controllability besides the exquisite sensitivity; see Penfield's homunculus for the motor cortex (Penfield & Rasmussen, 1950). If baseball were a sport in that pitchers throw from a body part with less controllability, such as the toes, that body part could not make the most of the delicate sense, if at all, for the controlled pitch. Namely, either or both conditions (1) and (3) cannot be satisfied. Another prerequisite is that the person who grips the ball understands that pitching a ball with accuracy and consistency is the pursuit of all baseball players (Fleisig et al., 2009; Glanzer et al., 2021; Kawamura et al., 2017; Kinoshita et al., 2017; Kusafuka et al., 2020; Manzi et al., 2021; Nasu & Kashino, 2021; Whiteside et al., 2016b). If this understanding is missing, the gripping experience will be a mere success in telling the seam direction with the fingertips; it will not sublimate into an Aha because condition (3) cannot be fulfilled. We also note that comprehending the S_4_ symmetry is not necessary for gaining confidence in truth; condition (4) is not requiring a level of mathematical rigor. The symmetry arguments can be of interest to those who are attentive to “the symmetries of things” (Conway et al., 2008). Discovering the rare S_4_ symmetry in a baseball can be an “Aha!” for chemistry course students who wish to have bonus points in the group theory class (Fuchigami et al., 2016), but this Aha is in problem-solving, not the “Aha!” initiated by the touch we are willing to nail down in this report.

**Figure 4.**
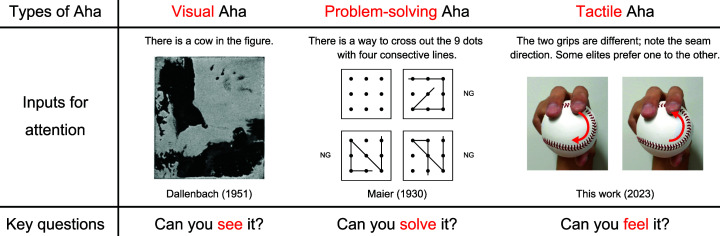
Tactile versus other types of Aha.

Finally, we wish to argue that the touch-driven “Aha!” is unique among the “Aha!”s widely studied over a century in psychological science. In 1907, Karl Bühler introduced the Aha experience (“Aha-Erlebnis” in German) into the literature (Bühler, 1907). Since then, investigations have begun into the insightful moments of Aha taking in various instances, such as when finding a solution to a problem (Kaplan & Simon, 1990; Maier, 1930), comprehending a joke or metaphor (Kounios & Beeman, 2014), or identifying a Dalmatian dog (Anderson, 2020) or Dallenbach's cow (Dallenbach, 1951) concealed in seemingly random ink blobs (Figure 4). The widely studied Aha's have been those driven by visual and verbal modalities. By contrast, the major sensation for the present anecdote comes not through the view of Figure 1 nor the verbal explanations, but through the touch. After directing attention to the seam direction, the key question to trigger the tactile Aha is, “Can you feel it?” The question differs from “Can you see/solve it?” asked, in some cases implicitly, in the tasks for investigating visual Aha's (Dallenbach, 1951) and those in problem-solving such as the 9-dot puzzle (Maier, 1930); see Figure 4. If the prerequisites are met, the trigger will ignite the tactile Aha. It may be worth investigating in future tasks whether the intensity of the tactile Aha correlates with the duration of the struggle for pitching a better 4-seam fastball without noticing the seam direction.

Concerning the investigations into cognitive processes in general, the tactile modality has been investigated in less detail than the other sensory modalities (Dehaene & Changeux, 2011; Gallace & Spence, 2008; Koch et al., 2016; Merrick et al., 2014). Recording brain activities in the event of Aha (Cui et al., 2021; Jung-Beeman et al., 2004; Kounios & Beeman, 2014; Qiu et al., 2010; Shen et al., 2018; Tik et al., 2018) triggered by gripping a baseball may provide new clues to the neural correlates of consciousness mediated by touch. Note, the ball is non-magnetic and is compatible with functional magnetic resonance imaging (fMRI) conducted in high magnetic fields.

In summary, we identified a new category in the “Aha!” experience, wherein the touch is the driving modality that intensifies the insight. A canonical example of this category is the novel “Aha!” that can occur when gripping a baseball, thanks to the elite pitchers disclosing their sense into the seam direction (Fujikawa, 2021; Yoshimi, 2021). Our finding provides a new path to investigate how sensations can appear in our brains through the intricate tactile modality. In addition, our symmetry analysis revealed the seam direction as a new degree of freedom in studying baseball aerodynamics and pitching mechanics, originating in the rare S_4_ symmetry figured out in this study. Last but not least, our study deepens the insight into throwing a baseball with accuracy and consistency, with more appreciation into our fingertips endowed with expert sensitivity and controllability.
